# CRISPR/dCas9-targeted H3K27me3 demethylation at the *CUC3* boundary gene triggers ectopic transcription and impacts plant development

**DOI:** 10.1016/j.isci.2025.112475

**Published:** 2025-04-21

**Authors:** Kateryna Fal, Salim El Khoury, Marie Le Masson, Alexandre Berr, Cristel C. Carles

**Affiliations:** 1 Grenoble Alpes University – CNRS – INRAE – CEA, Plant and Cell Physiology Lab, Bioscience and Biotechnology Institute of Grenoble, CEA, 17 rue des Martyrs, bât. C2, Grenoble Cedex 9 38054, France; 2 Institut de Biologie Moléculaire des Plantes du CNRS, Université de Strasbourg, 12 rue du Général Zimmer, Strasbourg Cedex 67084, France

**Keywords:** Epigenetics, Plant Biology, Plant development, Methodology in biological sciences

## Abstract

Chromatin modifications are deemed to correlate with gene expression patterns, yet their direct causal effect on transcription and cell fate remains unestablished. The H3K27me3 modification, highly conserved in eukaryotes, is strongly associated with the repression of developmental genes. Here, we establish the genuine function of H3K27me3 *in planta* by leveraging a CRISPR-dCas9-based epigenetic editing tool to specifically remove this methylation mark at the Arabidopsis *CUP SHAPED COTYLEDON 3* (*CUC3*) boundary gene. Targeted recruitment of the JMJ13 H3K27me3 demethylase to the *CUC3* locus induces ectopic transcription and gene expression patterns, leading to altered leaf morphology and meristem integrity. Combining molecular and phenotypic analyses, we thus establish evidence directly linking H3K27me3-mediated repression to developmental outcomes. Our study highlights locus-specific epigenetic editing as a powerful approach to dissect the functional impacts of histone modifications on transcription and morphogenesis, and provides a framework for unveiling the causal role of chromatin dynamics in plant developmental plasticity.

## Highlights

• CRISPR/dCas9-based tool to remove H3K27me3 at *CUC3*, an organ boundary gene in plants
• Targeted H3K27me3 demethylation induces ectopic transcription from the *CUC3* locus
• *CUC3* de-repression induces altered leaf shape and meristem development in Arabidopsis
• Locus-specific editing of H3K27me3 histone marks reveals their impact on morphogenesis

## Graphical abstract

**Figure:**
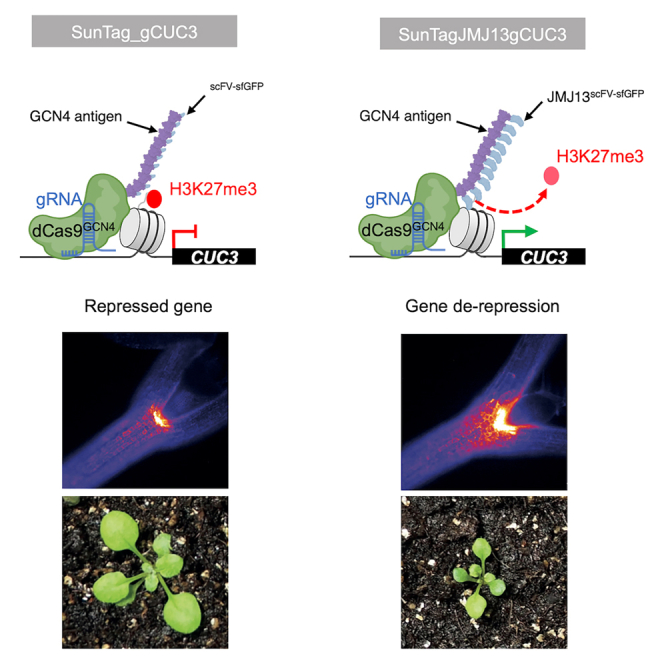

## Introduction

Considerable progress has been achieved in uncovering the genetic and epigenetic regulators of development in multicellular eukaryotes. Among them, key players are the chromatin complexes that bring post-translational modifications to histone tails and modulate access to DNA for the transcriptional machinery.^1^^,^^2^^,^^3^ In particular, the trimethyl mark deposited at Lysine 27 of Histone 3 (H3K27me3) is considered to control the dynamic regulation of key developmental genes, defining their spatial and temporal expression patterns and ensuring correct body plan establishment.^4^^,^^5^^,^^6^ This role for H3K27me3 has largely been deduced from the characterization of loss-of-function mutants in writers/erasers/readers, as well as from genome-wide profiling of marks and factor binding at the chromatin. Yet, such approaches are intricate due to the multifaceted interactions of the chromatin mark propagators, including their activity on non-histone substrates, their non-catalytic functions, and the functional specialization or redundancy of regulators within the same family, especially in plants.^4^^,^^7^^,^^8^^,^^9^^,^^10^ For these reasons, indirect functional studies have allowed drawing only limited and correlative conclusions on the relationships between H3K27me3 marks, transcriptional activity, gene expression, and body plan organization.

Therefore, to gain resolution on the genuine function of histone marks, approaches and tools for their direct editing have been developed.^11^ Manipulation of histone residues allowed revealing the key role of H3 methylations in animal and plant cell differentiation and specific developmental programs.^12^^,^^13^^,^^14^^,^^15^ In a prior study involving the editing of the H3K27 residue in *Arabidopsis thaliana*, we not only confirmed expected functions for the H3K27me3 mark but also discovered novel roles in cell fates, critical for tissue regeneration and plant architecture through stem tissue differentiation.^16^ While such global approaches have provided valuable insights, they affect the entire epigenome simultaneously, making it challenging to pinpoint the direct effect of a specific mark on a target gene.^11^ Hence, novel Clustered Regularly Interspaced Palindromic Repeats (CRISPR) - CRISPR associated proteins (CRISPR-Cas) derived tools have been developed for various model organisms, serving as a platform to tether an effector capable of modifying the expression or epigenetic marks at a precise genomic locus.^11^^,^^17^^,^^18^^,^^19^^,^^20^ These tools harbor a catalytically inactive (referred to as “dead”) form of Cas9 (dCas9), lacking endonuclease activity but retaining the ability to bind a single guide RNA (sgRNA).^21^ Thus far, dCas9 epigenetic editing tools have been more extensively assessed in animal cell cultures, with the aim to deposit or remove DNA methylation, histone acetylation, or methylation, albeit with mitigated degrees of success.^21^^,^^22^^,^^23^^,^^24^^,^^25^^,^^26^ In plants, only a limited number of studies have implemented CRISPR dCas9-based tools to manipulate epigenetic marks. These studies focused on editing DNA methylation,^27^^,^^28^^,^^29^ acetylation at H3K27,^30^^,^^31^ and methylation at H3K4^31^ and H3K9,^31^^,^^32^ primarily analyzing molecular effects on the epigenetic mark and gene expression, without delving into the developmental consequences.

Here we present a novel approach utilizing the CRISPR-dCas9 SUperNova (SunTag) system to manipulate for the first time the repressive H3K27me3 mark at the organ boundary *CUP-SHAPED COTYLEDON 3 (CUC3)* gene.

## Results

### Choice of the *CUP SHAPED COTYLEDON3* boundary gene as the target locus for the H3K27me3 edition strategy

**Figure 1:**
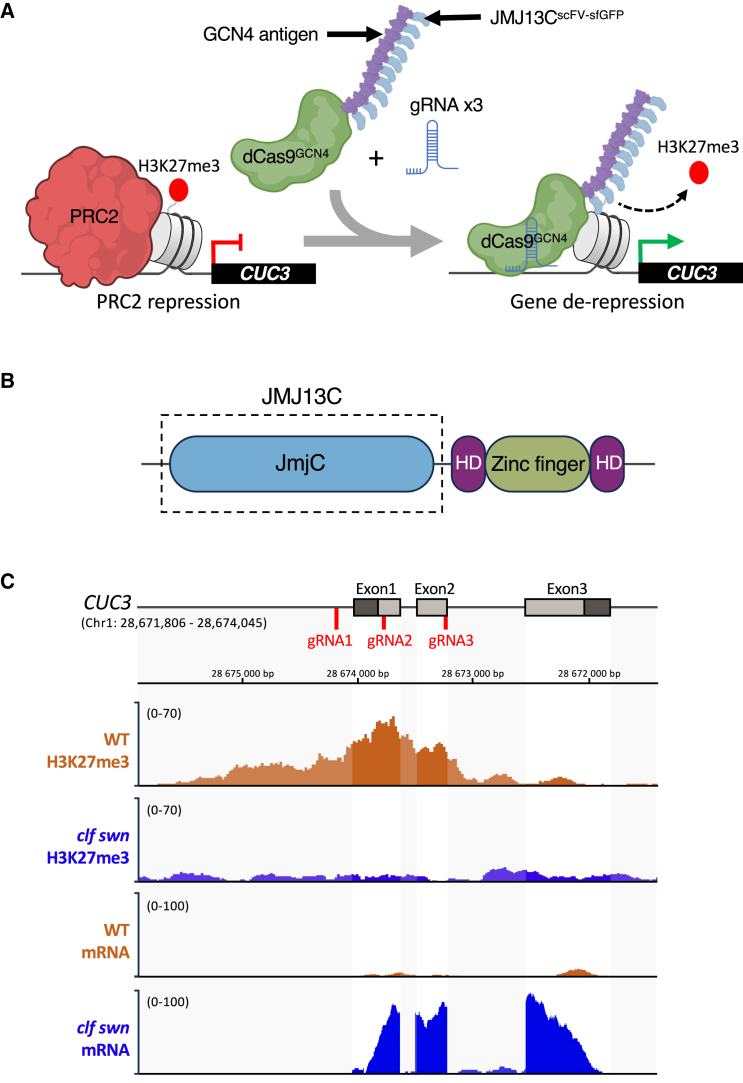
The dCas9-JMJ13^CUC3^ histone modification editor, a new tool designed to specifically remove H3K27me3 at *CUC3* (A) Schematic representation of the chromatin editing approach for targeted removal (dash-lined curved black arrow) of the repressive histone modification H3K27me3 (depicted as a red dot on the H3 histone tail) from the 5′ part of the *CUC3* gene region, using the dCas9-based tool with the Sun-Tag amplification system. The dCas9^GCN4^ construct can recruit up to ten copies of the chromatin-modifying module JMJ13C^scFV-sfGFP^ to target the *CUC3* regions via specific gRNAs. Violet bar: multimer of GCN4 antigens, present in 10 repeats; blue shapes: JMJ13 C-terminal domain, fused to the anti-GCN4 scFV (single-chain variable fragment) and sfGFP (Superfolder GFP). (B) Schematic representation of the Arabidopsis JMJ13 protein structure, containing the catalytic domain JMJC and the C4HCHC-type zinc finger domain.^36^ The dash-lined box outlines the protein region selected for use in this study. (C) Representation of the *CUC3* genomic region (AT1G76420), with the line marking the promoter and introns and the gray rectangles indicating the exons (dark gray delineates the 5′UTR and 3′UTR). ChIP-seq signals showing the H3K27me3 levels at this locus in 2-week-old seedlings is illustrated by the area highlighted in orange for the wild-type (WT) control and in blue for the double mutant of the PRC2 histone methyltransferases *CURLY LEAF (CLF) and SWINGER (SWN)*.^37^ Expression information, as captured by RNA-seq, is also informed for both genotypes.^37^ Red lines below the *CUC3* genomic region indicate positions of the three guide RNAs (gRNAs) designed in this study.

The rationale for selecting the specific mark-gene pair H3K27me3-*CUC3* is that H3K27me3 was reported to be a major determinant of tissue-specific expression patterns at the plant shoot apex,^33^ where the *CUC3* gene is differentially expressed, delimiting the boundary between the shoot apical meristem and the organ primordia.^34^ For this purpose, the Jumonji C-domain (JMJC) domain of the Arabidopsis JUMONJI13 (JMJ13) demethylase^35^ (Figures 1A and 1B) was integrated into the dCas9 SunTag system,^27^ allowing the recruitment of several effectors per locus, facilitated by an epitope-antibody amplification mechanism (Figures 1A and S1).

### Design and production of the dCas9-JMJ13^CUC3^ tool to manipulate the H3K27me3 mark at the *CUP SHAPED COTYLEDON3* developmental gene

Several JMJ domain proteins in plants are known to act as histone demethylases^38^^,^^39^^,^^40^^,^^41^ with three of them specifically targeting H3K27.^35^^,^^36^^,^^42^^,^^43^ Arabidopsis JMJ13, in particular, has been reported to contribute to photoperiod-dependent flowering regulation and self-fertility through the removal of histone methylation with high specificity toward repressive H3K27me3.^36^^,^^41^^,^^43^ Based on the reported structure of JMJ13, we selected and cloned the JMJC catalytic domain of Arabidopsis JMJ13 to be incorporated into the CRISPR-dCas9 system, with the aim of precisely removing H3K27me3 at the selected region. The dCas9 SunTag amplification system was chosen based on its successful application in previous reports for DNA methylation editing in plants.^27^^,^^28^^,^^29^

*CUP SHAPED COTYLEDON3 (CUC3)* gene encodes a NAC domain family transcription factor that (along with *CUC1* and *CUC2*) plays a pivotal role in shoot meristem initiation and maintenance, organ initiation and separation, leaf shape, and positioning of the carpel margin meristems.^34^^,^^44^^,^^45^^,^^46^^,^^47^^,^^48^^,^^49^^,^^50^ The expression of *CUC* genes is regulated through multiple pathways, including transcriptional control and post-transcriptional regulation by microRNAs (miRs) of the miR164 family for *CUC1* and *CUC2.*^45^^,^^51^^,^^52^^,^^53^^,^^54^^,^^55^ The expression of *CUC3*, that lacks the miRNA target site, is positively regulated by *CUC2*.^46^^,^^51^^,^^56^ In addition, the *CUC3* gene region exhibits enrichment in the repressive epigenetic mark H3K27me3 in leaf tissues as compared to shoot meristems,^33^ indicating the contribution of epigenetic mechanisms to the regulation of its expression (Figure 1C). We thus hypothesized that the targeted removal of the repressive H3K27me3 at the *CUC3* region would help to better understand the contribution of this epigenetic modification to gene expression regulation and serve as proof of concept for the editing of this chromatin mark.

We designed three sgRNAs, based on available data for H3K27me3 enrichment in Arabidopsis seedlings,^33^^,^^37^^,^^57^ to bring the dCas9-JMJ13 activity to the *CUC3* genomic region (Figure 1C). These sgRNAs were designed to target the promoter and proximal parts of the gene. Specifically, gRNA1 is positioned within the promoter region, gRNA2 near the transcription start site (TSS), and gRNA3 within the first exon of *CUC3*.

We hereinafter refer to the epigenetic editing tool developed in this study as the dCas9-JMJ13^CUC3^ tool. To assess its impact, the reporter line *pCUC3::CFP*^45^ was selected as the recipient for the dCas9-JMJ13^CUC3^ editing tool. This choice facilitates the monitoring of transcription from the *CUC3* promoter as well as expression from the *CUC3* endogenous locus.

Several independent transgenic lines were produced, carrying constructs with or without the JMJ13 catalytic domain, thereafter referred to as SunTagJMJ13gCUC3 and SunTag_gCUC3, respectively (SunTagJMJ13gCUC3: 41 primary transformants, 10 analyzed lines at the T2 generation, among which 4 were included in the study for analyses on the T3 and T4 generations; SunTag_gCUC3: 90 primary transformants, 10 analyzed lines at the T2 generation, among which 2 were included in the study for analyses on the T3 and T4 generations). The effects of the dCas9-JMJ13 tool on developmental features and target gene expression were deduced from analyses on the SunTagJMJ13gCUC3 plants in comparison to SunTag_gCUC3 and untransformed *pCUC3::CFP* (thereafter referred to as WT) plants.

### The dCas9-JMJ13^CUC3^ tool induces the developmental phenotypes characteristic of *CUP SHAPED COTYLEDON3* ectopic expression

**Figure 2:**
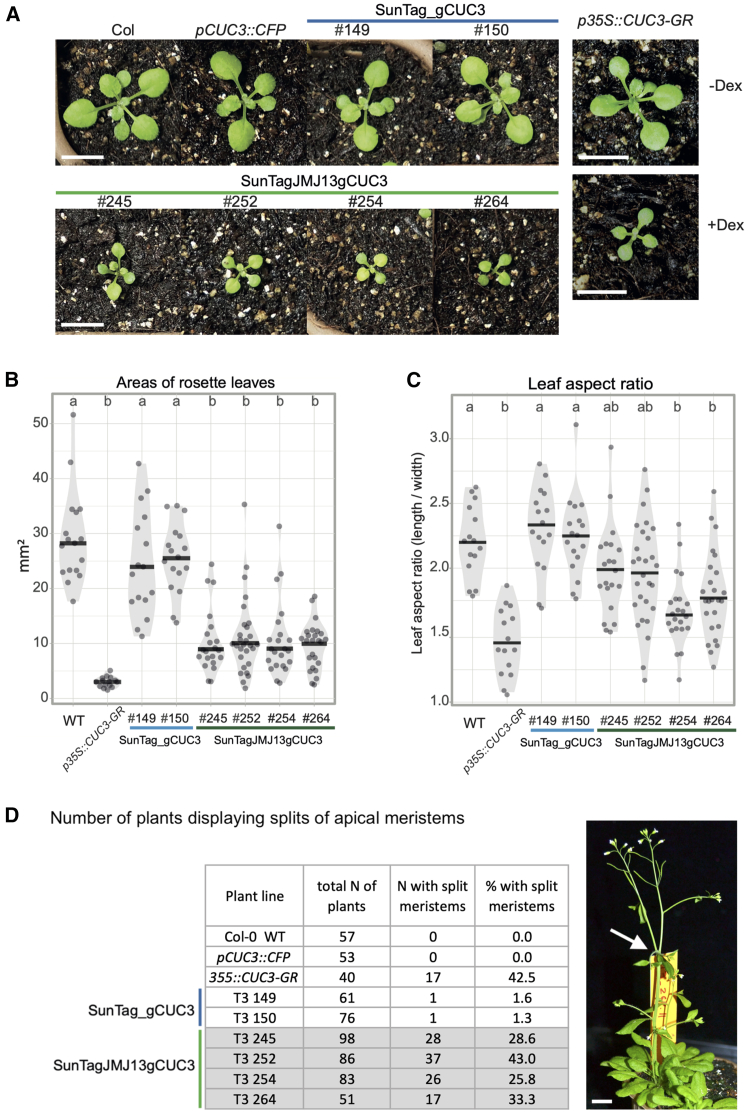
The dCas9-JMJ13^CUC3^ tool induces rosette phenotypes associated with *CUC3* ectopic expression (A–C) (A) Representative images of 16-day-old plantlets grown at 21°C under long-day conditions. The upper panel features (from left to right) plants from the wild-type Col-0 ecotype, the *pCUC3::CFP* (WT) line and two independent transgenic lines containing the dCas9 construct without the JMJ13 catalytic domain (SunTag_gCUC3). The lower panel features plants from four independent transgenic lines harboring the dCas9 construct with the JMJ13 catalytic domain (SunTagJMJ13gCUC3). The right panel displays images of plants from the inducible *p35S::CUC3-GR* line, grown on soil, in the absence (-Dex) or presence (+Dex) of dexamethasone. Scale bars: 1cm. Diagrams showing (B) the surface area (mm^2^) and (C) aspect ratio of leaves (length/width) for each genotype mentioned in (A). Sample size: *n* = 16, 15, 16, 17, 21, 28, 22 and 25 for WT, *p35S::CUC3-GR*, #149 and #150 (SunTag_gCUC3), #245, #252, #254 and #264 (SunTagJMJ13gCUC3), respectively. Black lines represent medians and dots represent values of individual samples. Letters indicate genotypes of the same statistical groups (Tukey pairwise comparison test, *p* < 0.05). (D) Table illustrating the number of plants displaying splits of the main stem (as pointed out by the arrow in the right-hand picture - example of a T3 plant from the SunTagJMJ13gCUC3 #264 line; more pictures can be found in Figure S3C), within three independently grown populations. The p*35S::CUC3-GR* plants were treated with Dex. N: number. Scale bar: 1cm.

Under long-day conditions, the plants of SunTagJMJ13gCUC3 lines displayed lower growth rates as compared to WT and SunTag_gCUC3 plants (Figure 2A). Specifically, for the four analyzed SunTagJMJ13gCUC3 independent lines, the areas of the rosette leaves are significantly smaller than those of the two independent SunTag_gCUC3 control lines (Figures 2B and S2). Additionally, the rosette leaves of dCas9-JMJ13 plants have an overall lower length-to-width aspect ratio than control plants (Figure 2C). These smaller rosette and rounder leaf phenotypes are similar to those, earlier reported, of plants conditionally overexpressing *CUC3* (*p35S::CUC3-GR* transgenic lines) and correspond well to the known functions of the CUC3 transcription factor as a growth repressor.^44^^,^^58^

SunTagJMJ13gCUC3 adult plants also display noticeable developmental phenotypes. Notably, we detected splits of the main shoot apical meristems in all four SunTagJMJ13gCUC3 lines, occurring with various frequencies (between 25.8% for line #254 and 43% for line #252) (Figures 2D and S3A). Some rare other defects, such as fasciated stems, were also observed (Figure S3A). Moreover, after final elongation, the SunTagJMJ13gCUC3 plants, on average, initiated a higher number of stems from the rosette and displayed a trend toward shorter overall inflorescence stem length (Figures S3B and S3C). While these traits presented some variability within plants of the same line and between independent lines, they consistently displayed a trend significantly different from the control lines (WT and SunTag_gCUC3). As a matter of fact, the ectopic expression of *CUC* genes has also been associated with an increase in branching.^54^

Together, these observations provide a good indication that the dCas9-JMJ13^CUC3^ tool leads to the ectopic de-repression of *CUC3*, likely as a result of the intended decrease in the repressive H3K27me3 mark. To verify this, we conducted further experiments on three of the SunTagJMJ13gCUC3 lines in comparison to SunTag_gCUC3 lines and a WT control, all in the *pCUC3::CFP* background.

### dCas9-JMJ13^CUC3^ leads to the activation of *CUP SHAPED COTYLEDON3* transcription within its expression territory and ectopically

**Figure 3:**
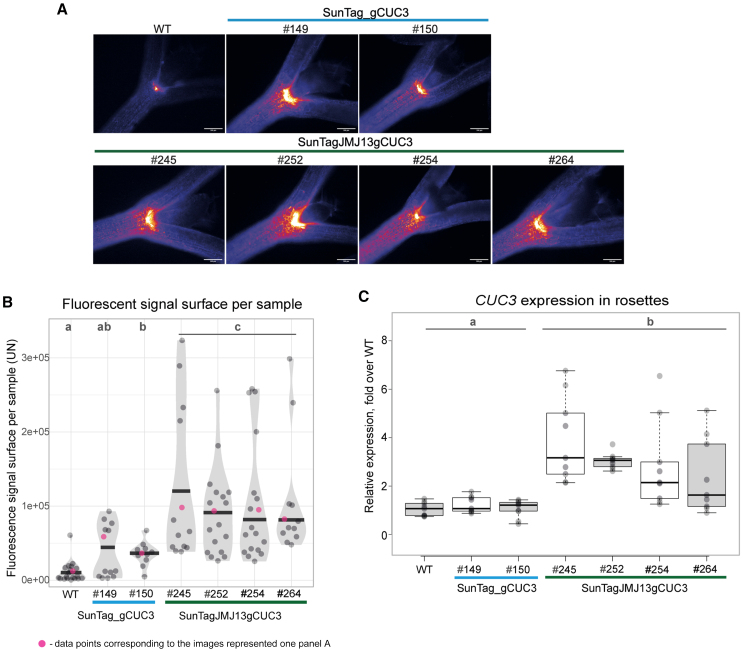
Transcription from the *pCUC3* promoter and expression of *CUC3* are induced in SunTagJMJ13gCUC3 lines (A) Representative fluorescence microscopy images of the 10-day-old seedlings visualising the CFP reporter expressed from the *CUC3* promoter (*pCUC3::CFP*). The upper panel displays the plants of wild-type plants and two independent transgenic lines that contain the dCas9 construct without the JMJ13 catalytic domain (SunTag_gCUC3). The lower panel displays the representative plant images of four independent transgenic lines that contain the dCas9 construct with the JMJ13 catalytic domain (SunTagJMJ13gCUC3). Scale bars: 200μm. (B) Violin plots illustrate the quantification of the fluorescent signal surfaces on individual microscopy samples (seedlings). Sample size: *n* = 19, 13, 11, 13, 18, 18, and 12 for WT, #149 and #150 (SunTag_gCUC3), #245, #252, #254 and #264 (SunTagJMJ13gCUC3), respectively. Black lines represent the median and the dots represent the values of individual samples; the samples are assembled in statistical groups by the Tukey pairwise comparison test. Letters indicate genotypes of the same statistical groups (p<0.05). (C) Boxplots representing the relative expression of *CUC3* in the seedlings of the control and test lines. *TUBULIN4 (TUB4)* was used as a reference gene for normalisation. Black lines represent the median and the dots represent values scored for individual seedlings. Letters indicate gennotypes of the same statistical groups (Tukey pairwise comparison test, *p* < 0.05).

We further analyzed the effects of *dCas9-JMJ13*^*CUC3*^ on its target transcription and expression in seedlings, using two distinct approaches. Firstly, the CFP fluorescent signal produced from the *pCUC3::CFP* construct was used for analysis of transcription from the *pCUC3* promoter. CFP signals were visualized by epifluorescence microscopy on 10-day-old seedlings from all test and control lines, and quantified from pictures taken on individual samples (Figures 3A, 3B, and S4A). While heterogeneity in signal intensity was present among the seedlings within each line, the quantification of the overall area covered by fluorescent signal showed that it was significantly more intense, as well as larger, in seedlings of the SunTagJMJ13gCUC3 lines compared to the SunTag_gCUC3 and WT lines. This indicates both a stronger transcriptional activity from the *pCUC3* promoter and a broader domain of expression within the seedling tissue. Second, to assess *CUC3* expression from the endogenous locus, we employed RT-qPCR, comparing rosettes from the SunTagJMJ13gCUC3 and SunTag_gCUC3 lines. The level of *CUC3* mRNA was increased from 2 to 7-fold depending on the plant and line (Figure 3C), consistent with results obtained from whole mount *in situ* hybridization (Figure S4B). Hence, the relative expression trends observed among lines, by RT-qPCR and whole-mount RNA *in situ* hybridization, were in agreement with the results of the *pCUC3::CFP* fluorescence analyses, and indicate a dCas9-JMJ13-induced de-repression of transcription at the *pCUC3* promoter and at the *CUC3* locus.

### Decreased level of H3K27me3 at *CUP SHAPED COTYLEDON3* correlates with its transcriptional reactivation

**Figure 4:**
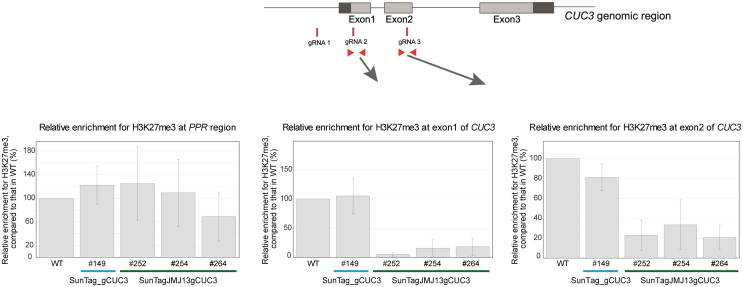
The dCas9-JMJ13^CUC3^ tool induces a reduction in the H3K27me3 mark abundance at the *CUC3* gene region, in SunTagJMJ13gCUC3 lines Histograms illustrating the relative enrichment of H3K27me3 at two regions of *CUC3*, depicted by the schematic drawing on the top, as detected by ChIP-qPCR. The *PPR* (AT5G55840) gene region was used as a negative control. The relative H3K27me3 enrichment was calculated as a fold change between the percentage of enrichment from the input, obtained after immunoprecipitation with the anti-H3K27me3 antibody, over that obtained with the anti-H3 antibody for the corresponding samples, and is represented relative to WT (set to 100). Each histogram bar corresponds to the mean value (the error bars indicate the standard deviation), calculated from of 3 biological repeats (for each repeat, the PCR quantification was performed with 3 technical replicates). The individual results of the 3 independent ChIP experiments can be visualised in Figure S5.

Finally, to assess if the dCas9-JMJ13^CUC3^-induced changes in *CUC3* expression were due to an expected, significant decrease in the H3K27me3 mark, we analyzed its abundance at the *CUC3* locus in seedlings, for all SunTagJMJ13gCUC3 transgenic lines that displayed robust phenotypes and effects on target gene expression. Using ChIP-qPCR, we detected that the amount of H3K27me3, reported to the amount of H3, was indeed lower in the SunTagJMJ13gCUC3 lines as compared to the control lines (WT and SunTag_gCUC3). This effect was the strongest within the first exon of *CUC3*, with a 5 to 10-fold decrease in H3K27me3 abundance, while the mark amount was reduced by 3–5-folds in the second exon (Figures 4 and S5). Interestingly, according to ChIP-seq data, the first exon is the region of the *CUC3* locus where H3K27me3 is most abundant (Figure 1C). Importantly, no significant decrease in H3K27me3 was detected in the SunTag_gCUC3 control, supporting the functionality (H3K27me3 demethylase effect) of the chosen JMJ13 catalytic domain when fused to the dCas9 SunTag system.

Finally, to evaluate the possibility of off-targeting to the *CUC3*-related genes *CUC1* and *CUC2*, we measured the expression of these two genes in the SunTagJMJ13gCUC3 lines via RT-qPCR (Figure S6A). We found that neither *CUC1* nor *CUC2* exhibited expression changes similar to those observed for *CUC3* (see Figure 3C for comparison). Moreover, to assess for an unintended dCas9-HA-GCN4 binding at *CUC1* and *CUC2* in the SunTagJMJ13gCUC3 lines, we performed ChIP-qPCR experiments using an antibody directed against HA. The data show that, while amplifications at the *CUC3* locus from the IP samples reach 23%–61% of that from the inputs, amplification values for *CUC1* and *CUC2* ranged from 0.6 to 7.2% of the inputs (Figure S6B), indicating that the targeting is highly specific to *CUC3*.

## Discussion

Here, we have reported the use of a CRISPR dCas9-based system employing the JMJ13 catalytic domain to selectively remove the repressive H3K27me3 mark and thereby manipulate transcription from the organ frontier gene *CUC3* in Arabidopsis.

Our results reveal that the inflicted decrease in the repressive epigenetic mark at targeted regions results in the derepression of *CUC3* within plant tissues and is associated with distinct developmental phenotypes. This comprehensive dataset provides a compelling proof-of-concept, seamlessly bridging molecular insights to developmental evidence. It thus validates a valuable approach to dissect the roles of individual histone marks in the regulation of chromatin structure and transcription dynamics in plants, with an ultimate readout on cell fate decisions.

With our characterization of dCas9-JMJ13^CUC3^, precise chromatin editing tools proved instrumental in assessing whether chromatin marks can be primary determinants of gene expression and cell differentiation. They could likely be pushed further toward inducible systems for more precise temporal post-perturbation analyses, thereby allowing the exploration of changes in the nucleus and chromatin structure, cross-talks between epigenetic marks, and shifts in transcription kinetics. These tools promise new insights into chromatin-mediated regulation, supporting future studies of transcriptional plasticity and plant developmental processes at unprecedented resolution.

### Limitations of the study

When designing sgRNA for epigenetic editing, one must be aware that the gRNA design principles were initially optimized for genome editing, and not necessarily for chromatin modification. The number and location of sgRNA to be used for epigenetic editing largely depend on the genomic profile of the target mark to be modified (spread throughout the locus, enriched at the TSS, …). For histone PTMs such as methylation and acetylation, particularly when they are dispersed across a locus, several sgRNA may be required to achieve sufficient coverage and efficient modification. Furthermore, while the *dCas9-JMJ13*^*CUC3*^ construction has a significant overall impact on *CUC3* expression, we observed heterogeneity in response between cells within the same tissue. This heterogeneity, as revealed by *in situ* CFP fluorescence imaging, may be attributed to factors such as differential chromatin accessibility, varying dCas9 availability, or intrinsic cellular states within the tissue. Such variations could lead to inconsistent expression changes across cells and ultimately affect developmental outcomes, even within plants of the same line. To address these limitations, future approaches may benefit from incorporating tissue-specific, developmental stage-specific, or inducible promoters. These enhancements would allow for more refined spatial and temporal control over epigenome editing interventions, improving the consistency and precision of responses. For instance, inducible systems activated by heat shock or specific hormones could enable precise timing of chromatin mark removal, helping to dissect the causal relationships between chromatin dynamics, gene expression, and developmental processes.

## Resource availability

### Lead contact

Further information and requests for resources and reagents should be directed to and will be fulfilled by the lead contact, Christel Carles (Christel.carles@univ-grenoble-alpes.fr).

### Materials availability

Plasmids generated in this study (corresponding to the dCas9-JMJ13^CUC3^ tools) are available upon request.

### Data and code availability

Sequence data from this article can be found in the GenBank/EMBL libraries under the following accession numbers: *CUC3* (At1g76420), *TUB4* (At5g44340), and *PPR* (At5g55840).

This article does not report a high-throughput dataset nor original code. Any additional information required to re-analyze the data reported in this article is available from the lead contact upon request.

## Acknowledgments

We thank Anne-Marie Boisson, Dila Cetin, Adrien Galeone, Emilien Krempf, Alizée Musso, and Mirko de Vivo for help with plant culture, selection, and characterization of transgenic lines.

This work was supported by the 10.13039/501100001665Agence Nationale de la Recherche (ANR-18-CE20-0011-01, PRC project REWIRE to C.C.C. and A.B.), the Grenoble Alliance for Cell and Structural Biology (ANR-10-LABX-49-01), and the CBH Graduate School of UGA (ANR-17-EURE-0003).

## Author contributions

Conceptualization, C.C.C. and K.F.; methodology, C.C.C, K.F., and A.B.; formal analysis, C.C.C. and K.F.; investigation, K.F., S.E.K., M.L.M., and C.C.C.; writing – original draft, K.F. and C.C.C.; writing – review and editing, C.C.C. with help from A.B., S.E.K., and K.F.; funding acquisition, C.C.C. and A.B.

## Declaration of interests

The authors declare no competing interests.

## STAR★Methods

### Key resources table

**Table:** 

REAGENT or RESOURCE	SOURCE	IDENTIFIER
**Antibodies**		

anti-trimethyl-H3K27	Merck	Cat #07-449; RRID: AB_310624
anti-H3	Agrisera	Cat #AS10710; RRID: AB_10750790

**Bacterial and virus strains**		

*Escherichia coli* DH5α	N/A	N/A
*Agrobacterium tumefaciens* EHA105	N/A	N/A

**Chemicals, peptides, and recombinant proteins**		

KpnI restriction enzyme	Thermo Scientific™	Cat #ER0522
MauBI restriction enzyme	Thermo Scientific™	Cat #ER2081
BsiWI restriction enzyme	Thermo Scientific™	E Cat #R0851
Sodium hypochlorite solution	Merck	Cat #1056142500, CAS: 7681-52-9
Triton X-100	Sigma-Aldrich	X100-500ML
Plant agar	Sigma-Aldrich	A4550-1KG
MS basal salt mixture, powder	Sigma-Aldrich	Cat #M5524
Hygromycine B	Merck	Cat #H0654-500MG
Agarose	Roth	3810.4
Proteinase K	Merck	1245680500
GoTaq DNA Polymerase	Promega	Cat #M784A
GoTaq reaction buffer	Promega	Cat #M792A
dNTPs	Thermofisher	R0186
Formaldehyd7e (36% solution stabilized in 9% methanol)	VWR	Cat #20909.290
Glycine	Merck	Cat #G8898
Tris	Promega	H5135
HCl 37%	Sodipro	CL0003101000PE
EDTA	Promega	H5032
Sodium chloride	Euromedex	Cat #1112.A
DNase I	Ambion	Cat #AM1907
RNase inhibitor	Merck	3335399001
Dextran	Sigma-Aldrich	31389-500G
Ficoll PM400	Sigma-Aldrich	F4375-100G
Sucrose	Sigma-Aldrich	16104-2.5KG
PMSF	Thermofisher	36978
Protease inhibitor	Thermofisher	A32965 et A32955
Protein A beads (without salmon sperm DNA)	Merck	16–661
Sodium acetate	Merck	W302406

**Critical commercial assays**		

RNeasy Plant Mini Kit	Qiagen	Cat #74904
ezDNase SuperScript IV VILO	Fisher Scientific	Cat #15523145
POWER SYBR GREEN PCR	Fisher Scientific	Cat #10658255
Reaction Minelute Kit	Qiagen	Cat #28206

**Deposited data**		

Sequence data		*CUC3* (At1g76420), *TUB4* (At5g44340), *PPR* (At5g55840).

**Experimental models: Organisms/strains**		

Columbia-0 accession (Col-0)	NASC	Cat #N1093
*CUC3* (At1g76420)	GenBank accession number	AF543195
*TUB4* (At5g44340)	GenBank accession number	AY059075
*PPR* (At5g55840)	GenBank accession number	NM_001345162
*pCUC3::CFP3* (Col-0)	Gonçalves et al.^46^	N/A
*p35S::CUC3-GR* (Col-0)	Serra & Perrot-Rechenmann^58^	N/A
SunTag_gCUC3 (Col-0)	This study	lines #149, #150
SunTagJMJ13gCUC3 (Col-0)	This study	lines #245, #252, #254, #264

**Oligonucleotides**		

See Table S1	Eurofins	N/A

**Recombinant DNA**		

SunTag dCas9 plasmid	Addgene	Cat #117168
gRNA cassette	GenScript, custom-synthesized	N/A
dCas9-JMJ13^CUC3^ plasmid	This study	N/A
dCas9-0^CUC3^ plasmid	This study	N/A

**Software and algorithms**		

CHOPCHOP	Open source	http://chopchop.cbu.uib.no/
Cas-Offinder	CRISPR RGEN Tools, open source	http://www.rgenome.net/cas-offinder/
Fiji	ImageJ, open source	https://imagej.nih.gov/ij/
Zeiss LSM	Zeiss, open source	https://www.embl.de/eamnet/html/body_image_browser.html
Rstudio software	RStudio Team (2020), open source	http://www.rstudio.com/

### Experimental model and subject details

#### Plant line details

All *Arabidopsis thaliana* plant lines used in this study are in the Col-0 ecotype, which is the wild-type accession used for all experiments (control and transgenic lines). The reporter and inducible lines were described previously: *pCUC3::CFP*^46^ and *p35S::CUC3-GR*.^58^ The following lines are described in this study: SunTag_gCUC3 (independent lines #149 and #150) and SunTagJMJ13gCUC3 (independent lines #245, #252, #254 and #264). Primers used for genotyping are indicated in Table S1.

#### Plant culture & selection of transgenic lines

All plants were cultured in growth chambers, under long-day conditions, 16 h/8 h light/dark period, at 21°C, following a stratification period of at least 3 days at 4°C, in obscurity. Seeds for *in vitro* culture were sterilised in 50% bleach and 0.1% Triton for 8 min and rinsed three time with sterile water. For the selection of transgenic lines, the seeds of transformed plants were germinated and grown for 10 days on Murashige-Skoog (MS) plates containing Hygromycin B (Merck H0654-500MG). Resistant plants were transferred to soil and genotyped with the primers listed in Table S1. Lines with a single insertion locus were brought to the T3 generation for further characterization.

### Method details

#### Cloning and generation of transgenic lines

sgRNA design was performed using the CHOPCHOP tool (http://chopchop.cbu.uib.no/, Repair profile prediction^59^) combined with Cas-Offinder (http://www.rgenome.net/cas-offinder/) and TAIR blast tools for verification of off-target effects. Selected sgRNA sequences are as follows, with in brackets, the predicted efficiency (should be > 50 for recommended use) and the predicted number of off-targets: gRNA1 AGAGACTCACTCCCCAGGTGAGG (60.3; 0), gRNA2 GATGTGTTAAGCGAACTCGCCGG (69.6; 0), and gRNA3 CACTTAGTCTTGAGGCCACGTGG (66.4; 0). The gRNA cassette was custom-synthesised by GenScript (www.genscript.com) and inserted into into the SunTag dCas9 plasmid (Addgene Plasmid #117168) using the KpnI and MauBI restriction enzymes (Thermo Scientific, ER0522 and ER2081 respectively). The JMJ13 catalytic domain was amplified with the primers listed in Table S1 and cloned into the SunTag dCas9 plasmid using the BsiWI restriction enzyme (Thermo Scientific, ER0851). The final construct allows the production of (i) a dCas9 fusion to 10 copies of the short epitope GCN4, (ii) a superfolderGFP-JMJ13 effector domain combination fused to a single-chain variable fragment (scFV) antibody directed against GCN4, and (iii) three sgRNAs complementary to the *CUC3* genomic sequence (Figure S1).

#### Plant phenotyping

The procedures for quantitative phenotype characterisation were performed on plants of the T3 and T4 generations.

Detection of the CFP expression in the tissues was performed on 10-day-old MS plate-grown seedlings. Images were acquired using the Zeiss Imager.M2 microscope (20× and 40× objective) with the Axiocam 503.

The size of rosettes was assessed from images of 15-day-old plants using the Fiji software,^60^ by drawing circles that touched the extremities of 3 rosette leaves on each plant. The areas and aspect ratio of rosette leaves were measured by outlining the contour of the third true leaf on individual plants within the population.

The inflorescence stem length and quantity of side branches were quantified on plants with fully elongated main stems after all flowers were opened.

#### Whole-mount *in situ* hybridization

The abundance of *CUC3* transcript in tissues of generated transgenic lines was estimated with the whole mount *in situ* hybridization performed on 10-day-old MS plate-grown seedlings. The probe used was amplified from Arabidopsis inflorescence cDNA using the primers listed in Table S1 (earlier tested in^49^) and labeled using the Roche DIG RNA Labeling Mixture (11277073910). The tissue fixation and hybridization procedures were performed as described in Rozier et al.^61^ Prepared samples were imaged with a Keyence Digital Microscope VHX-5000.

#### Plot preparation and statistical analysis

Plots of all presented datasets were prepared using the Rstudio software (RStudio Team (2020), http://www.rstudio.com/). The Tukey’s range test was used to make the pairwise comparisons of means from independent samples.

#### Gene expression analyses

Expression of the *CUC* genes in the generated lines was verified by RT-qPCR. RNA was extracted from rosette leaves of 15-day-old plants and purified using the Qiagen RNeasy Plant Mini Kit (Cat. No./ID: 74904). After DNase treatment (ezDNase SuperScript IV VILO, ThermoFisher, Cat. No. 11756050), first strand cDNA synthesis was performed from 2μg of total RNA using SuperScript IV VILO (ThermoFisher, Cat. No. 11756050). Relative transcript abundance was measured using the SYBR Green Master Mix (POWER SYBR GREEN PCR, Fisher Scientific, 10658255) on a CFX Connect BioRad Real-Time PCR System. Gene-specific primers used for amplification are listed in Table S1.

#### Chromatin immunoprecipitation

Chromatin fraction was isolated from 10-day-old seedlings following the procedure described in Engelhorn et al.^57^ The antibodies used were anti-trimethyl-H3K27 (07–449 Merck), anti-H3 (AS10710 Agrisera) and anti-HA (H3663 Sigma). Reverse-crosslinked samples were purified using the Qiagen Reaction MinElute Kit (#28206) with an elution volume of 20μL. The procedure was carried out on samples collected and prepared from 3 independently grown plant populations. Immuno-precipitation was performed on chromatin extracts, using either the anti-H3K27me3 antibody, the anti-H3 antibody or the anti-HA antibody. The ChIP-qPCR for selected target regions was performed as described above for the RT-qPCR, with 3 technical replicates, using the primers listed in Table S1. The H3K27me3 enrichment was calculated relative to that obtained after immunoprecipitation with the anti-H3 antibody for each corresponding sample. The HA enrichments at the *CUC* loci in the immunoprecipitated samples were calculated from the absolute amplification values, as percentage of amplifications obtained from the input.

### Quantification and statistical analysis

For all experiments, detail of statistical tests used for diagrams and error bars on histograms are indicated in the figure legends.
