# Supplementary material for: CRISPR/dCas9-targeted H3K27me3 demethylation at the CUC3 boundary gene triggers ectopic transcription and impacts plant development
Source: iScience. 2025 Apr 21;28(5):112475. doi: 10.1016/j.isci.2025.112475 (PMC12153052; doi:10.1016/j.isci.2025.112475)
Supplement: Document S1. Figures S1–S6 and Table S1 [file mmc1.pdf]

iScience, Volume *28*

Supplemental information

CRISPR/dCas9-targeted H3K27me3 demethylation at the *CUC3* boundary gene triggers ectopic transcription and impacts plant development

Kateryna Fal, Salim El Khoury, Marie Le Masson, Alexandre Berr, and Cristel C. Carles

Supplementary Figure 1

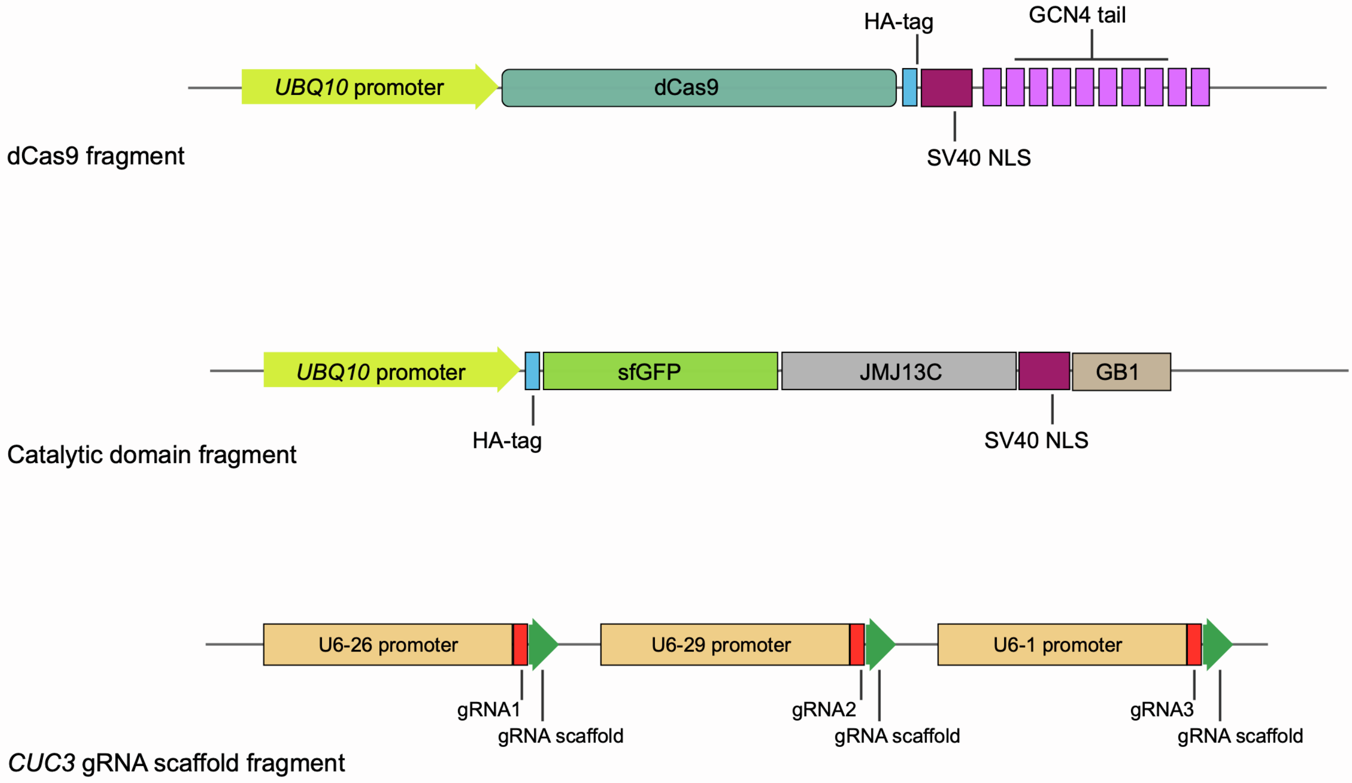

Supplementary Figure S1. Schematic illustrating the three modules of the SunTag construct. The dCas9GCN4 module consists of dCas9 fused to a tail made of 10 copies of the GCN4 epitope and a triple SV40 NLS, whose expression is controlled by the UBQ10 promoter. The JMJ13CscFv-sfGFP module consists of the catalytic domain of JMJ13 fused to scFv-sfGFP and a GB1-REX NLS (NLS sequences present in the SunTag construct reported in27, whose expression is also controlled by the UBQ10 promoter. The gRNA module consists of three sequential expression cassettes with gRNAs whose expression is controlled by independent U6 promoters (U6-26, U6-29 and U6-1).

Supplementary Figure 2

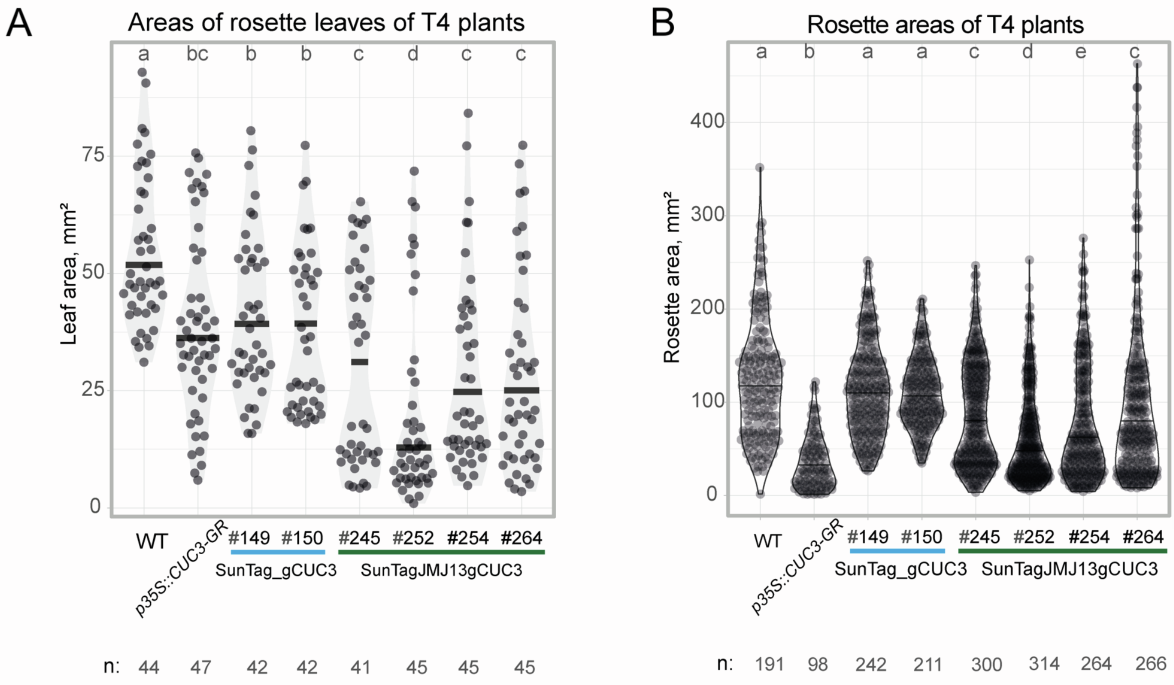

Supplementary Figure S2. The dCas9-JMJ13CUC3 tool induces rosette phenotypes associated with CUC3 ectopic expression. Diagrams showing the average leaf (A) and rosette (B) surface areas (mm2) for the plants of WT, p35S::CUC3-GR, SunTag_gCUC3 and SunTagJMJ13gCUC3 genotypes. The surface areas of the third leaf were measured in plants from two independent T4 populations with the total sample size: n = 44, 47, 42, 42, 41, 45, 45 and 45 for WT, p35S::CUC3-GR, #149 and #150 (SunTag_gCUC3), #245, #252, #254 and #264 (SunTagJMJ13gCUC3), respectively. The rosette area measurements were acquired on plants from two independent T4 populations with the total sample size: n = 190, 91, 319, 210, 305, 314, 255 and 269 for WT, p35S::CUC3-GR, #149 and #150 (SunTag_gCUC3), #245, #252, #254 and #264 (SunTagJMJ13gCUC3), respectively. Black lines represent medians and dots represent values of individual samples. Letters indicate significant differences (Tukey pairwise comparison test, P<0.05).

Supplementary Figure 3

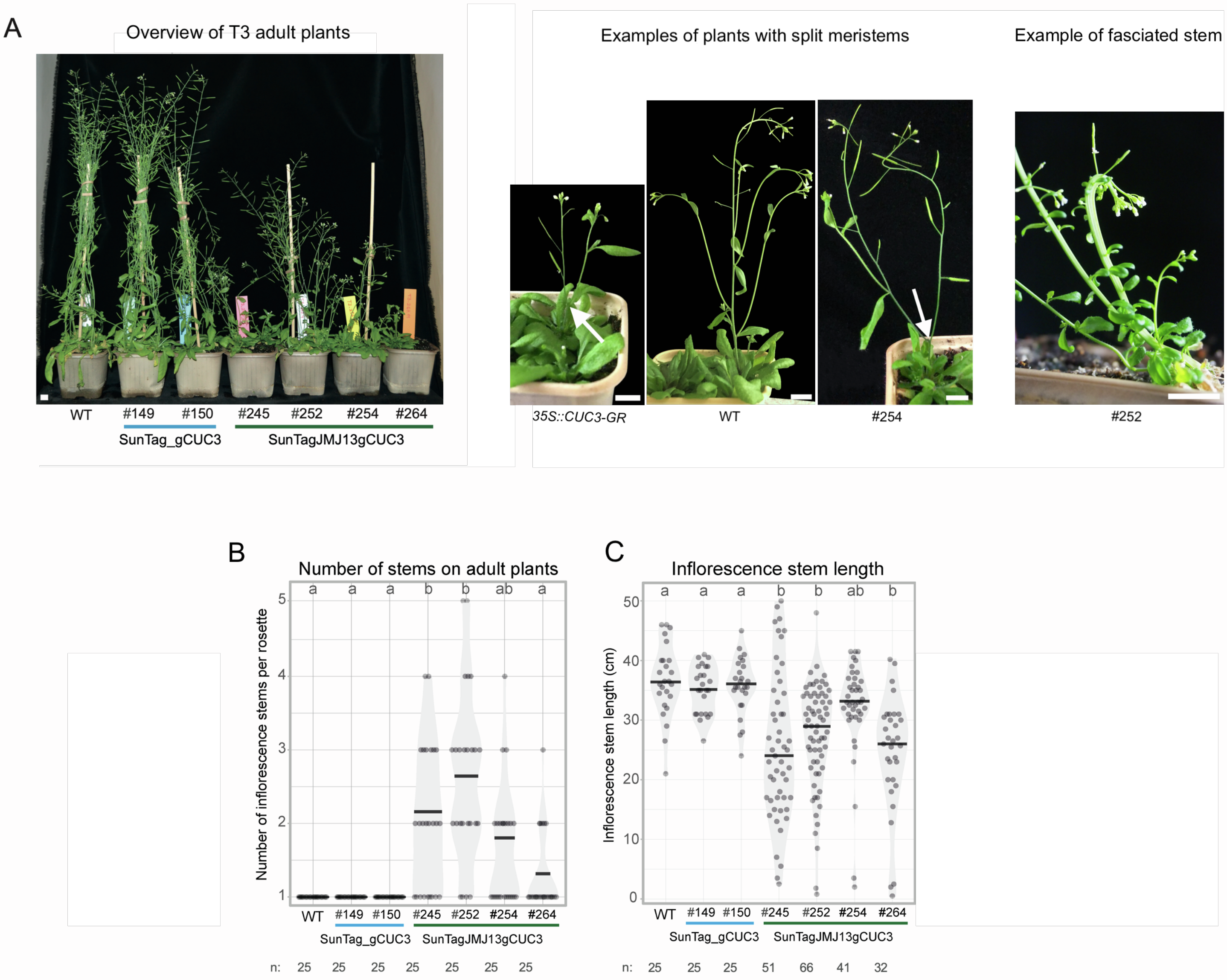

Supplementary Figure S3. Adult plant phenotypes, associated with the dCas9-JMJ13CUC3 tool and CUC3 ectopic expression. (A) Representative image of the adult plants of (from left to right) WT line, two independent transgenic lines containing the dCas9 construct without the JMJ13 catalytic domain (SunTag_gCUC3) and four independent transgenic lines caring the dCas9 construct with the JMJ13 catalytic domain (SunTagJMJ13gCUC3). All pictured plants belong to the simultaneously sown populations, grown at 21°C under long-day conditions. Examples of split meristem and fasciated stem phenotypes are also shown for the p35S::CUC3-GR line and SunTagJMJ13gCUC3 #254 and #252 lines. Arrows point to splits of stems. Scale bars: 1cm. (B) Diagram displaying the average number of inflorescence stems on the plants from each of the genotypes, mentioned in (A) with the total sample size of n = 25 for all the genotypes. (C) Diagram illustrating the average maximal inflorescence stem length for the plants from each of the genotypes, mentioned in (A) with the sample size of n = 25, 25, 25, 51, 66, 41, and 32 for WT, #149 and #150 (SunTag_gCUC3), and #245, #252, #254 and #264 (SunTagJMJ13gCUC3), respectively. All phenotype quantification measurements for B and C were acquired on plants from two independent T4 populations. Black lines represent medians and dots represent values of individual samples. Letters indicate significant differences (Tukey pairwise comparison test, P<0.05).

Supplementary Figure 4

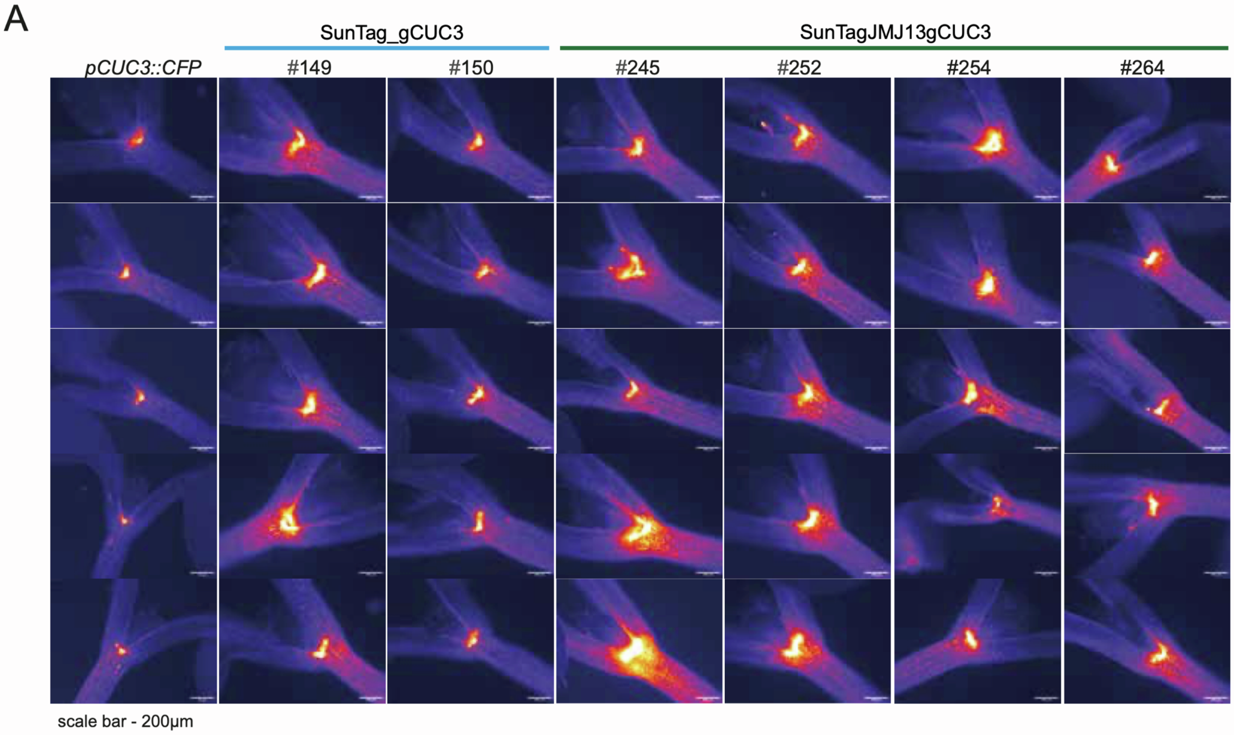

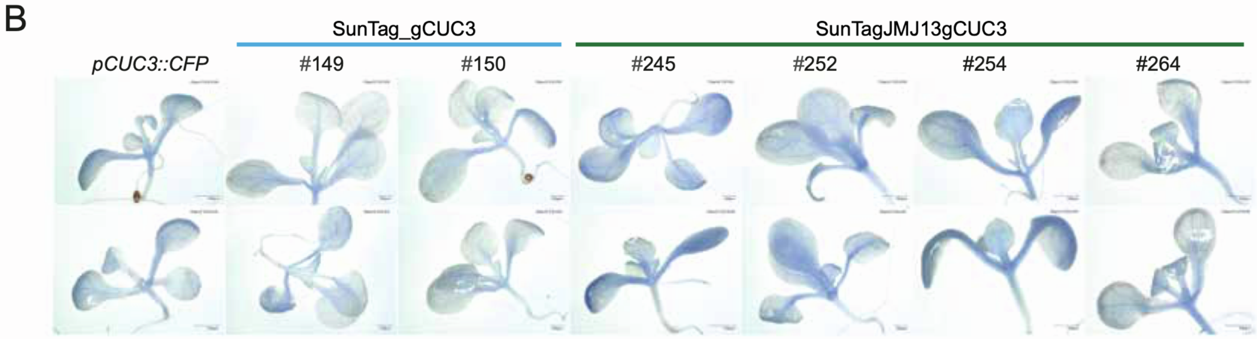

Supplementary Figure S4. Representative images of fluorescence microscopy and whole mount in situ hybridization for CUC3 promoter activity and transcript accumulation, respectively. (A) Representative fluorescence microscopy images of the 10-day-old seedlings, visualizing the CFP reporter expressed from the CUC3 promoter (pCUC3:CFP). The columns from left to right display the plants of wild type and two independent transgenic lines that contain the dCas9 construct without the JMJ13 catalytic domain (SunTag_CUC3) followed by four independent transgenic lines that contain the dCas9 construct with the JMJ13 catalytic domain (SunTagJMJ13gCUC3). Scale bars: 200um. (B) Representative images of whole mount RNA in situ hybridization of CUC3, performed on 10-day-old seedlings of the same lines as in (A).

Supplementary Figure 5

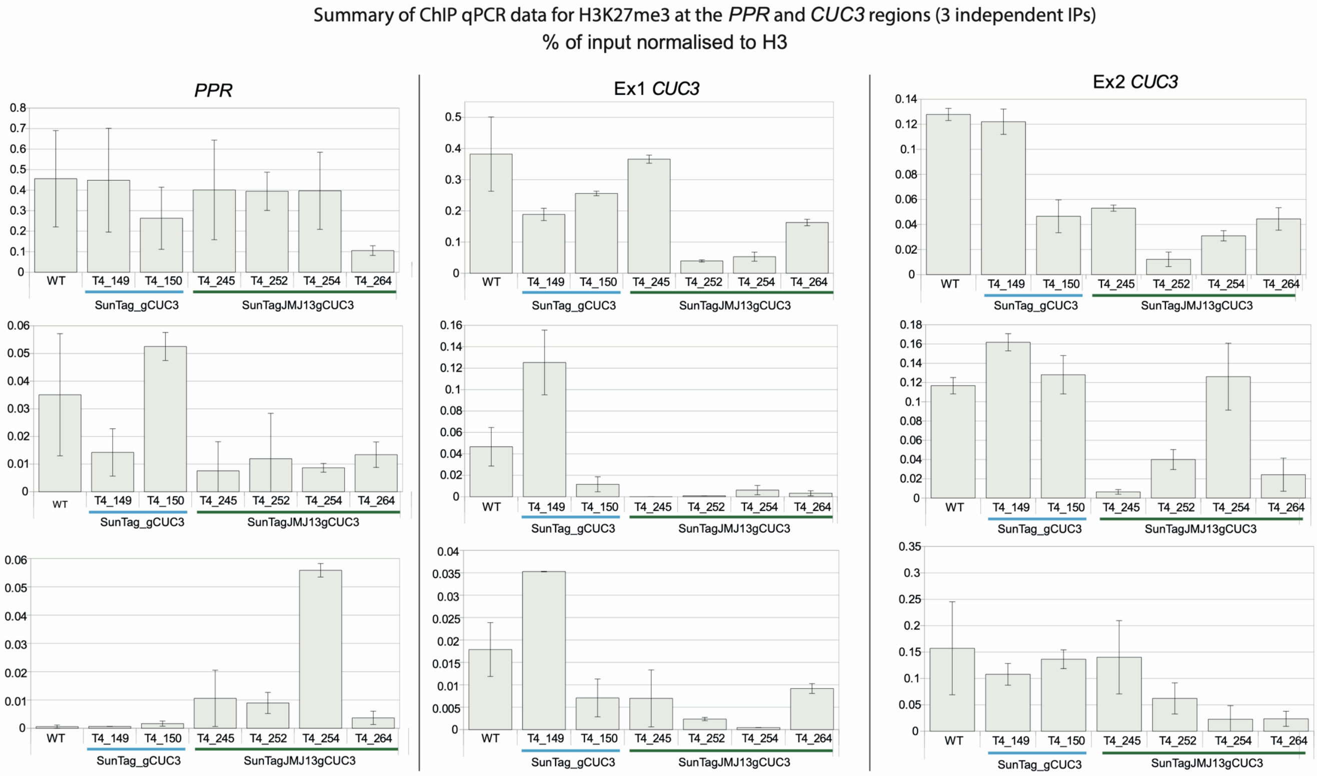

Supplementary Figure S5. The dCas9-JMJ13CUC3 tool induces reduction in the H3K27me3 mark abundance at the CUC3 gene region, in SunTagJMJ13gCUC3 lines. Histograms illustrating the relative enrichment for H3K27me3 at two regions of CUC3, depicted by the schematic drawing in Figure 4, as detected by ChIP-qPCR. The PPR (AT5G55840) gene region was used as a negative control. The relative H3K27me3 enrichment was calculated as a fold change between the percentage of input enrichment obtained after immunoprecipitation with the anti-H3K27me3 antibody, over that obtained with the anti-H3 antibody for the corresponding samples, and is represented relative to WT (set to 100). The individual results of the three independent ChIP experiments are presented in three independent graphs organised in column, with the mean values (and standard deviation) for each histogram calculated from three technical replicates. Only one line (#245) out of the four tested did not display consistent changes between replicates.

Supplementary Figure 6

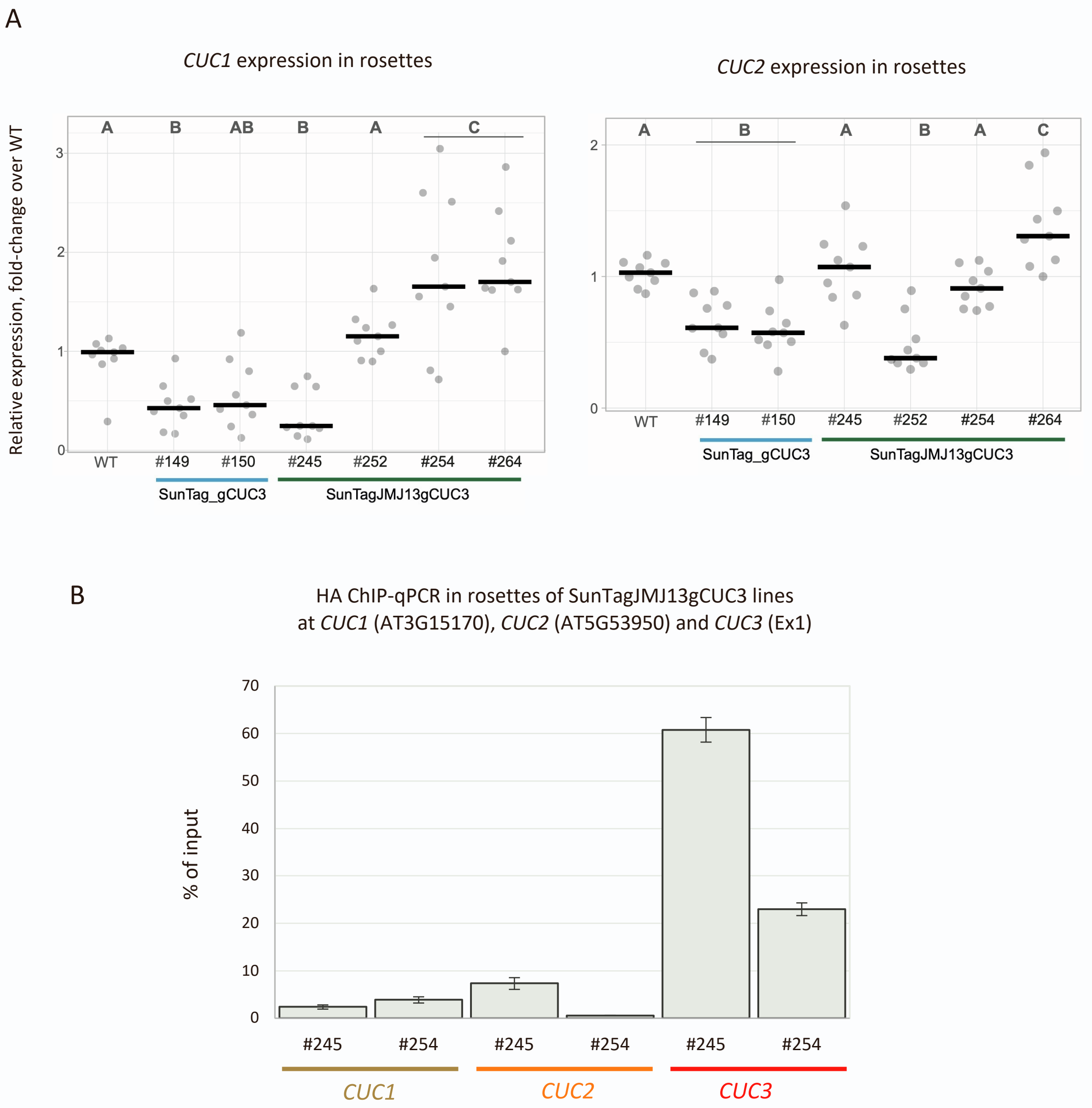

Supplementary Figure S6. The dCas9-JMJ13CUC3 tool does not induce changes in *CUC1* and *CUC2* expression similar to that observed for *CUC3*, and is not found to be enriched at the chromatin of the corresponding genes, as compared to *CUC3*. (A) Plots representing the relative expression of *CUC1* or *CUC2* in the seedlings of the control and test lines (fold-change over the WT control). *TUBULIN4* (*TUB4*) was used as a reference gene for normalisation. Black lines represent the median and the dots represent values scored for individual seedlings. Letters indicate significant differences (Tukey pairwise comparison test, P<0.05).
(B) Histograms illustrating the relative enrichment for the HA tag at *CUC1* to *CUC3* genes, as detected by ChIP-qPCR. The amplification signal was quantified from 2 sets of samples obtained from independent ChIP preps, with three technical triplicates. Mean values are given in % of input. Error bars represent standard deviation.

Supplementary Table 1

Primer name	Target	Primer sequence
BsiWI_JMJ13_noATG_F	JMJ13 catalytic domain	CGTACGGCGGAAAGGAGGATCTGCTTG
BsiWI_JMJ13_noATGnoZnF_R	JMJ13 catalytic domain	CGTACGGACTATGGTTCCATATGTATTC
JMJ13seq2 R	*transgene genotyping*	CTTTATCATCAGAATCCCACTCAG
sfGFPend_F	*transgene genotyping*	GGTCCTTCTTGAGTTTGTAAC
SunTag_GB1 R	*transgene genotyping*	CTACCACCACCTTCGGTTAC
gRNA-CUC3 – R	*transgene genotyping*	GGGAGTGAGTCTCTAATCAC
TUB4 QPCR-R	*TUBULIN 4*	AGGGAAACGAAGACAGCAAG
TUB4 QPCR-F	*TUBULIN 4*	CTGTTTCCGTACCCTCAAGC
CUC1QRTF	*CUC1*	TCTGCCGGTTCTGCAATTG
CUC1QRTR	*CUC1*	CATCGGTATGAGCAGCAGAGTT
CUC2QRTF	*CUC2*	ATGGCGGAGACAGCCAATATCTT
CUC2QRTR	*CUC2*	GACTTTGCGGAGAAGGTAATG
qCUC3_F	*CUC3*	GAGTATCGCCTCGAAAACGA
qCUC3_R	*CUC3*	TGTGTTGTTGAGAGTGAATGGTT
CUC3 situF	*in situ*	ACTCTACGATGATCCACACCA
CUC3 situ RT7	*in situ*	TGTAATACGACTCACTATAGGAGCATGTGAAGAGGTCCAGG
CUC3_IP Ex1 F2	*CUC3*_ ChIP	AGATGATGCTTGCGGTGGAA
CUC3_IP Ex1 R2	*CUC3*_ ChIP	AATGAGCTCTTCGTCCGTCG
CUC3_IP Ex2 F3	*CUC3*_ ChIP	GAGGAGGACAGCTTGTTGGG
CUC3_IP Ex2 R3	*CUC3*_ ChIP	GAATGGTCGTTTTCGAGGCG
AT5G55840_F	*PPR* _CHIP	CTTGTGGCGAGAGAGAGCAA
AT5G55840_R	*PPR* _CHIP	TTCGAAGTTTTGACAGCGCC

Supplementary Table S1. Information on primers used in this study.
